# A systematic approach to deep learning-based nodule detection in chest radiographs

**DOI:** 10.1038/s41598-023-37270-2

**Published:** 2023-06-21

**Authors:** Finn Behrendt, Marcel Bengs, Debayan Bhattacharya, Julia Krüger, Roland Opfer, Alexander Schlaefer

**Affiliations:** 1 Institute of Medical Technology and Intelligent Systems, Hamburg University of Technology, 21073 Hamburg, Germany; 2 Jung Diagnostics GmbH, 22335 Hamburg, Germany

**Keywords:** Medical research, Engineering

## Abstract

Lung cancer is a serious disease responsible for millions of deaths every year. Early stages of lung cancer can be manifested in pulmonary lung nodules. To assist radiologists in reducing the number of overseen nodules and to increase the detection accuracy in general, automatic detection algorithms have been proposed. Particularly, deep learning methods are promising. However, obtaining clinically relevant results remains challenging. While a variety of approaches have been proposed for general purpose object detection, these are typically evaluated on benchmark data sets. Achieving competitive performance for specific real-world problems like lung nodule detection typically requires careful analysis of the problem at hand and the selection and tuning of suitable deep learning models. We present a systematic comparison of state-of-the-art object detection algorithms for the task of lung nodule detection. In this regard, we address the critical aspect of class imbalance and and demonstrate a data augmentation approach as well as transfer learning to boost performance. We illustrate how this analysis and a combination of multiple architectures results in state-of-the-art performance for lung nodule detection, which is demonstrated by the proposed model winning the detection track of the Node21 competition. The code for our approach is available at https://github.com/FinnBehrendt/node21-submit.

## Introduction

Chest radiographs (chest x-rays) are the most common radiological examination in clinical practice as they offer high diagnostic value, low radiation dose, and cost-effectiveness^1,2^. Among the wide variety of pathologies that can be diagnosed from chest x-rays, an important objective is to detect pulmonary lung nodules. These Pulmonary nodules can be an indication of lung cancer, a major reason for death worldwide^3^. Therefore, an early detection of nodules in chest x-rays is of high clinical importance^4,5^.

Detecting nodules in chest x-rays is challenging for radiologists, which is reflected in low inter- and intra-observer agreement as well as highly variable detection sensitivities that are reported in the literature^4,6,7^. A reason for this is seen in the modality itself, as signal characteristics of x-rays make it hard to distinguish overlapping structures and thus to identify nodules, placed behind certain anatomical structures. As a consequence, typically, a follow up computer tomography scan is requested if nodules are detected in x-rays^8^. This comes with the cost of time-intensive examinations and an increased radiation by a factor of 75 to 900^9,10^. Therefore, it is of high importance to achieve a high sensitivity and simultaneously reduce the number of false positive nodule detections in chest x-rays.

Computer-Aided Diagnosis (CAD) systems have been developed over the last few decades to assist radiologists in detecting and diagnosing diseases in chest radiographs, including pulmonary lung nodules. For this purpose, traditional methods involving manual feature extraction^11–13^, threshold-based methods^14^, active shape models^15^ and machine learning-based techniques like SVMs^16^ have been proposed. Recently, deep learning-based Convolutional Neural Networks (CNN) replaced these traditional approaches due to their ability to automatically extract high-level features from raw data, such as images, without the need for hand-crafted feature engineering. With the availability of large-scale data sets of chest radiographs^17–19^, deep learning approaches have been shown to achieve high levels of accuracy for global, image-level pathology and nodule screening^20–25^. However, the additional localization of nodules remains a challenging task. Several local methods have been proposed to address this issue, relying on patch-based classification^26,27^, semi-supervised activation maps from CNNs^28–30^ or supervised segmentation networks^31^. Commercial CAD systems are also available and clinical validation studies have shown that these systems can improve the nodule detection performance of radiologists considering both classical machine learning^5^ and deep learning-based approaches^32–34^. While customized CNN-based architectures are commonly used in the research field of nodule detection in chest x-rays, object detection methods optimized for generic images have proven their ability to perform well on benchmark data sets such as Common Objects in Context (COCO)^35–39^. Some of these methods have also been evaluated for nodule detection in chest x-rays, and have demonstrated promising results^40–48^. Nevertheless, object detection methods require localization information on nodules, which means that annotated data sets are necessary for training. Unfortunately, these data sets are limited and much smaller compared to the generic image data sets due to the expensive annotation process. As a result, the lack of large-scale publicly available data sets for training, evaluating, and benchmarking deep learning solutions for nodule detection in chest x-rays has been a major obstacle. Besides the high risk for overfitting on small data sets, this leads to a large-variety of deep learning-based solutions for nodule detection, evaluated on individual data sets which makes it hard to compare their performance and to distill the most promising approaches for nodule detection in chest x-rays. To address this issue, the Node21 competition^49^ was created. Its aim is to encourage participants to develop automated approaches with high performance for detecting nodules in chest x-rays. The competition provides large data sets with ground truth annotations from radiologists, enabling fair comparison and evaluation of the different approaches. The participants’ solutions are ranked based on their detection performance on an unseen test set, which replicates the clinical application scenario and emphasizes the importance of generalization.

A high performing approach for such real-world problems is typically not achieved by using a single model architecture that shows strong individual performance on specific test data sets. Instead, a careful analysis of the problem at hand and adjusting the approach to address problem-specific challenges are required. Furthermore, the tuning of various complementary model architectures is beneficial as ensembling is known to improve performance^50–52^.

Therefore, in this work we systematically study and identify two major challenges in the application of state-of-the-art object detection algorithms to the task of nodule detection in chest x-rays.

First, we pinpoint the absence of large-scale data sets for training as a hurdle. Even though there exist large-scale data sets of chest x-rays^17–19^, only a small part of the data sets contain nodules and even less reliable annotations from domain experts are available. In contrast, for generic images, large-scale data bases exist with reliable bounding box annotations. One approach to counteract small data set sizes is to apply transfer-learning. It is common practice to use models that are pre-trained on large scale data bases of generic images such as ImageNet^53^ or COCO. However, the domain of these data bases significantly differs from the domain of chest x-rays, and there is low evidence that the algorithms pre-trained on the image domain of Imagenet or COCO would transfer well to the target domain of chest x-rays^54^.

Second, we recognize that the available medical data sets often show a heavy class imbalance, where x-rays containing nodules are less frequent than their nodule-free counterparts. Deep learning models tend to favor the prediction of the majority class without proper re-balancing of the data which impedes the generalization of the models. Especially for object detection algorithms, class imbalances are problematic as the majority of possible bounding boxes often corresponds to the background class^55^. This problem is reinforced when applying object detection algorithms to the field of nodule detection, as high image-level class imbalances are inherent to publicly available data sets where only a fraction of chest x-rays contain annotated nodules, while the remaining x-rays exclusively contain the background class.

Having identified hurdles in applying deep learning-based object detectors to the task of nodule detection, we systematically evaluate different strategies to address them with the goal of improving the nodule detection performance.

First, we investigate the use of transfer learning with images from different source domains to account for the overall small training set sizes. Transfer learning is a widely used method when training deep learning-based approaches. Various studies have investigated supervised or unsupervised pre-training techniques, either from natural image domains^54^ or domains that closely resemble the target domain, such as chest x-rays^56,57^. Many studies focus on pre-training the feature extraction backbone of deep learning architectures. In contrast to that, our study employs pre-trained weights from object detection models that are specifically trained in an end-to-end supervised fashion on the VinDR^58^ chest x-ray data set (published by the Vingroup Big Data Institute). In addition, we investigate the impact of using pre-trained weights from the COCO object detection data set and training from scratch to determine which strategy yields the most significant benefits.

Second, we address the issue of class imbalance by exploring the use of generated nodules to augment the training data distribution. Specifically, we introduce nodules at random locations within the lung area of nodule-free chest x-rays. Similar approaches have been successfully employed in previous works, where RetinaNet has been trained solely on augmented chest x-rays^46^ or the nodule detection performance of RetinaNet has been improved by adding generated radiographs with nodules to the existing training data^59,60^. In this study, we examine the impact of nodule generation in the healthy background class samples to balance the data distribution and compare its effectiveness to oversampling the less frequent class, i.e., chest x-rays with nodules.

Lastly, we evaluate, rank and compare various object detection algorithms that show state-of-the-art performance on benchmark data sets of generic images for the task of nodule detection in chest x-rays. For our final nodule detection pipeline, we apply our collective insights and train four nodule detection algorithms, apply transfer learning and reduce the class imbalance by generated artificial nodules. Furthermore, we utilize techniques like data augmentation and stochastic weight averaging^61^ to achieve better generalization. We exploit that the different models learn complementary features from the chest x-rays and combine the different approaches to develop a well-generalizing ensemble.

Overall, we present a systematic approach to achieve state-of-the-art performance for nodule detection in chest x-rays. Rather than inventing entirely new techniques or model structures, we pinpoint the essential steps to achieve robust performance on real-world data. Our approach is systematic and well-grounded, based on a thorough analysis and comparison of the most effective techniques and methodologies and provides valuable insights to the field of nodule detection in chest x-rays. By applying our strategy, we are able to outperform all competing solutions in the Node21 competition and secure first place in the detection track.

## Methods

**Figure 1:**
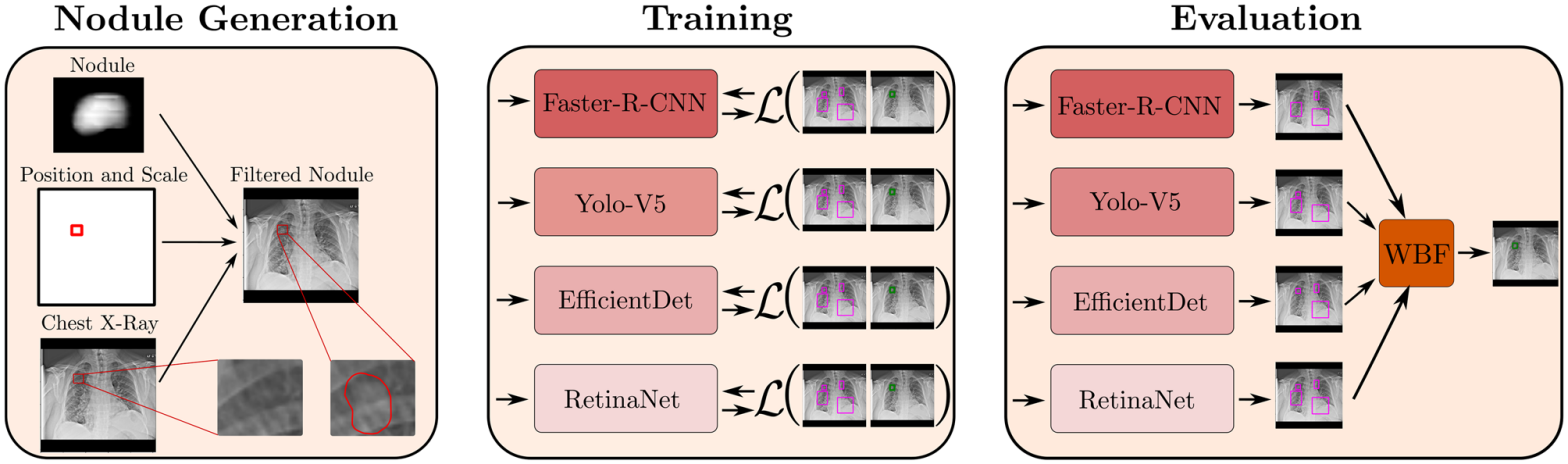
Schematic visualization of our deep learning pipeline for nodule detection. Left: Nodule generation process. A nodule is embedded in a nodule-free chest x-ray at a given position and scale. Middle: Training of multiple model architectures, independently of each other. Right: Evaluation and ensembling of the trained models. The predictions of each model are merged via weighted box fusion (WBF)^62^ which results in one aggregated prediction for all models.

In this section we provide details of data handling, training strategies, and hyper-parameters. An overview of our approach is provided in Fig. 1.

### Data set

For our experiments we use the Node21 competition data set. The data set consists of 4882 frontal chest radiographs that origin from four different public data sets (JRST^63^ (N=242), PadChest^18^ (N=1680), Chestx-ray14^17^ (N=1804), Open-I^19^ (N=1218)). All x-rays are revised and annotated by radiologists. The majority of the radiographs (N=3748) are free of nodules while 1134 radiographs show at least one nodule (1476 nodules in total). The annotation protocol of the Node21 challenge includes annotations of solitary, solid or subsolid nodules. Clusters of more than three nodules are filtered out and only nodules with a diameter of $6\,mm$ to $30\,mm$ are included in the data set. For the evaluation of the challenge submissions, two additional test sets are used by the organizers. One experimental test set $\mathscr {D}_{exp}$ (N=281) on which participants have a limited number of evaluations to test intermediate results and one final test set $\mathscr {D}_{final}$ to evaluate the final submission. In strong contrast to the training data, x-rays with nodules are more frequent than nodule-free x-rays in the experimental test set $\mathscr {D}_{exp}$. For the final test set $\mathscr {D}_{final}$, no details regarding the data set size or class distribution are provided. Furthermore, the radiologists have access to a computer tomography scan of the same patient for $\mathscr {D}_{exp}$ and $\mathscr {D}_{final}$ during the annotation process.

Since the provided test and training data originates from different data sources, a reliable evaluation strategy is required to account for the expected variance across the different data sets. Therefore, first, we partition the provided data set into training data (N=4532) and an additional test set $\mathscr {D}_{add}$ (N=350). We use $\mathscr {D}_{add}$ to evaluate intermediate development steps of our approach. To achieve a balanced test set with a variance similar to the training distribution, and similar class distribution as $\mathscr {D}_{add}$ we take an equal number of x-rays with nodules and x-rays without nodules from each public data set. For JRST, PadChest and Chest x-ray, we use 50 x-rays from each class respectively, while for the Open-I data set we only use 25 x-rays due to the low number of x-rays with nodules. Furthermore, we apply a 5-Fold cross-validation to the remaining data for hyper-parameter tuning. To ensure a similar class distribution across the folds, we sample the individual training and validation sets in a stratified fashion grouped by patients. A summary of all available training and test sets is provided in Table 1. After model tuning and selection, we merge the additional test set $\mathscr {D}_{add}$ to our training set to train the models for the final submission with all available data.

**Figure 2:**
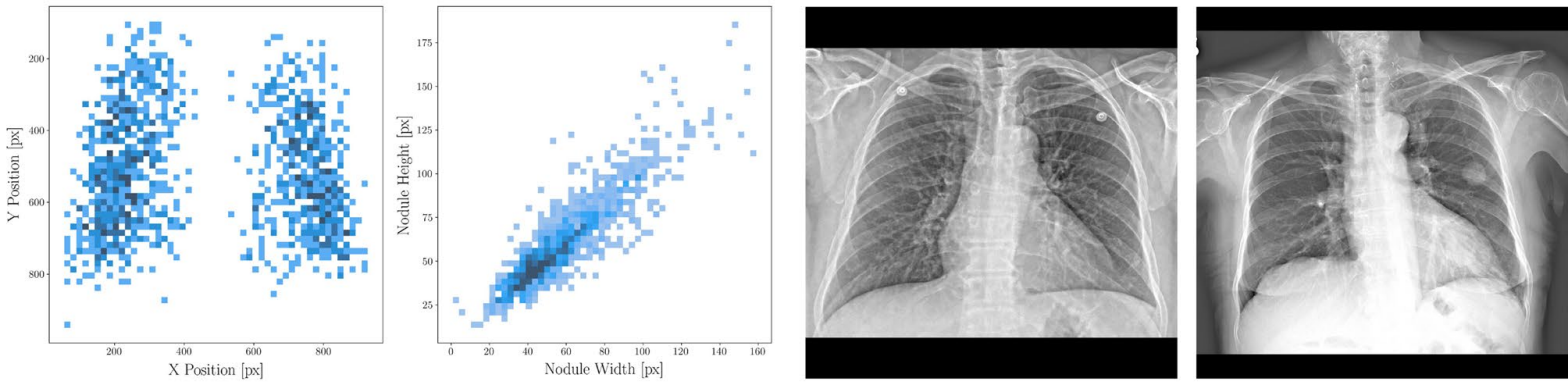
Analysis of bounding boxes in the data. From left to right: Position of all bounding boxes on the x and y axis height plotted against the width of all bounding boxes, respectively, exemplary x-ray without nodules, exemplary x-ray with one nodule.

Exemplary x-rays of the data set as well as a bounding box analysis regarding position and shape is provided in Fig. 2.

**Table 1:** Summary of the data sets with their respective number of samples and the fraction of x-rays containing nodules (positives). After sampling the additional test set, we apply a 5-fold cross-validation to the data set. For the training- and validation set, representative numbers of one fold are reported.

Data set		Number of samples	Fraction positives (%)
Training set	$\mathscr {D}_{train}$	3626	23
Validation set	$\mathscr {D}_{val}$	906	23
Add. test set	$\mathscr {D}_{add}$	350	50
Exp. test set	$\mathscr {D}_{exp}$	281	59
Final test set	$\mathscr {D}_{final}$	Unknown	Unknown

### Pre-processing

We follow the pre-processing strategy of the Node21 challenge: First, homogeneous border regions are removed. Second, energy-based normalization of x-ray intensity values is applied as proposed in^64^. Third, lung fields are segmented by a convolutional neural network and the x-rays are cropped to the segmented lung fields. Finally, all x-rays are resized from their original resolution to a resolution of $1024 \times 1024\,$px with bilinear interpolation, and padded to preserve the aspect ratio. For models that require an input resolution other than $1024 \times 1024\,$px, we further resize the x-rays to the desired input resolution.

### Deep learning models

Our general approach is to use an ensemble of different object detectors to leverage all individual benefits, exploit complementary features and to build a well-generalizing model. We include four different models to our final ensemble, namely Faster-R-CNN^36^, RetinaNet^37^, EfficientDet-D2^38^ and YoloV5^39^. In the following section, the architectures are presented and explained briefly. For a more detailed description, we refer to the referenced original publications.

**Figure 3:**
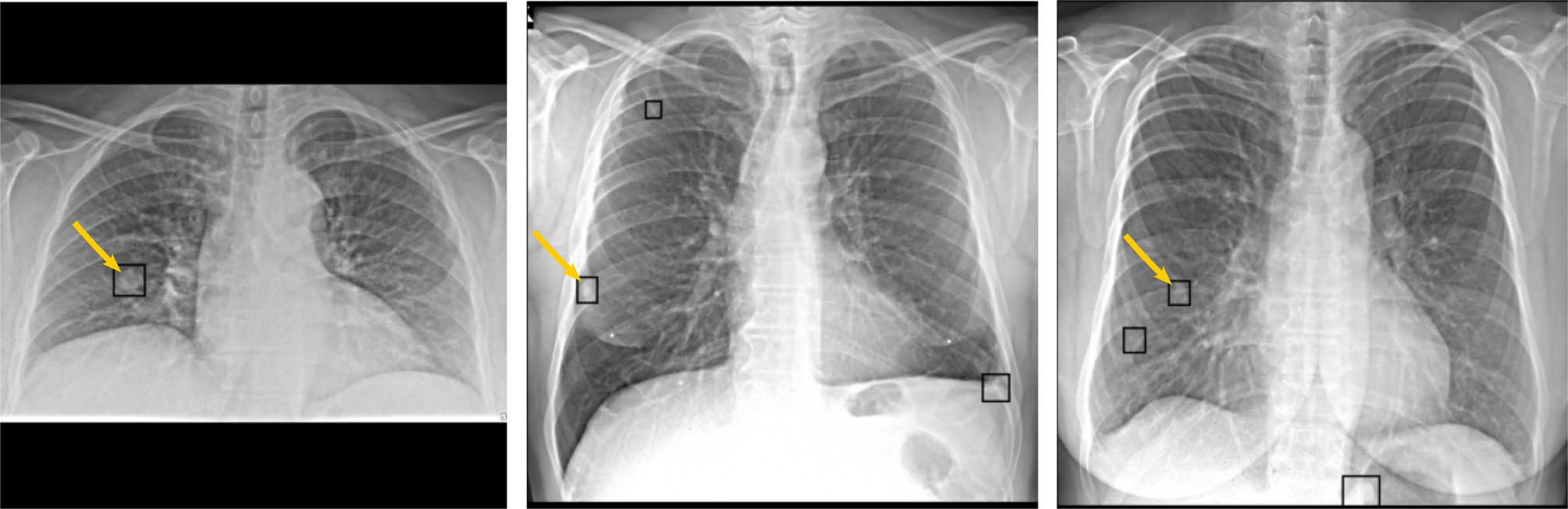
Left: Correctly predicted nodule. Middle and Right: Correctly predicted nodule together with false positive predictions. Yellow arrows indicate the groundtruth location of nodules.

The goal of each model is to predict pairs of bounding box coordinates and a score that corresponds to the probability of seeing a nodule in the respective bounding box. Exemplary predictions are shown in Fig. 3. All models are implemented in Pytorch.

#### Faster-R-CNN

Faster-R-CNN is a two-stage object detector where first region proposals are extracted from an input image and in a second step, these regions of interest are classified and the bounding box predictions are refined^65^. Faster-R-CNN emerged from two earlier network versions, namely R-CNN^65^ and Fast-R-CNN^66^. The main development of Fast-R-CNN and subsequently Faster-R-CNN is the improved and more efficient choice of region proposals for the respective object to detect^36,66^. To predict the class and position of bounding boxes, first, feature maps are extracted by a CNN backbone. Next, a convolutional region proposal network is used to predict the initial regions of interest in the feature maps. Finally, the bounding boxes and classification scores are predicted for each region of interest by a fully connected network.

#### RetinaNet

RetinaNet^37^ is a single-stage object detector that predicts bounding boxes and classification scores in an end-to-end fashion. Thereby, the input image is fed into a feature pyramid backbone CNN that outputs feature maps at multiple scales. From each pyramid level, the features are fed to two subnetworks, a box regression subnetwork for bounding box prediction and a classification subnetwork for classification. A core principle of RetinaNet is the use of focal loss for all predicted bounding boxes. Hereby, more importance is given to bounding boxes that are hard to predict by scaling the loss of each bounding box prediction^37^.

#### EfficientDet-D2

EfficientDet^38^ is a model family of single-stage object detectors. The key concepts of EfficientDets are weighted bi-directional feature pyramid networks (BiFPN) that are an advancement to the feature pyramids used in RetinaNet. Instead of fusing the features in a top-down fashion, in BiFPNs feature-levels are fused in a bi-directional fashion and with unequal, learned weights. As for EfficientNets^67^, an architecture search is conducted where the depth, width and input resolution is scaled for different EfficientNet variants. For each variant of EfficientDet, the respective EfficientNet variant is used as backbone network.

In our experiments, we use EfficientDet-D2 with an EfficientNet-B2 backbone.

#### YoloV5

Yolo, short for “You only look once” is a family of one-stage object detectors. Developing from YoloV1 to YoloV5, a variety of network structures, training procedures and specific post-processing strategies are adapted to efficiently improve the detection performance. The backbone of YoloV5 consists of a custom cross stage partial network^68^, called CSPDarkNet53. The extracted features are processed by a feature pyramid network with pathway fusion that aims for bi-directional fusion of feature levels^69^. A key concept of the Yolo family is extensive data augmentation and post-processing, integrated to the YoloV5 pipeline^39^. For our experiments, we chose YoloV5x as model architecture.

### Ensembling and post-processing

To obtain a high performance model for our final submission, we ensemble the predictions of Faster-R-CNN, RetinaNet, EfficientDet-D2 and YoloV5. Additionally, to become robust to different nodule sizes, we use two different input resolutions for the YoloV5 model, namely $640 \times 640$ px and $1024 \times 1024$ px. For each model, we train 5 and ensemble versions from different folds of the data set by performing a five-fold cross-validation approach. We post-process all bounding box predictions by first applying non-maximum-supression (NSM). Hereby, if two bounding boxes overlap by an intersection over union (IOU) greater than 20%, the box with the lower prediction score is removed. Finally, we ensemble all model predictions by using weighted box fusing and remove all bounding boxes with a predicted score below 0.1. In weighted box fusion, overlapping bounding boxes are fused by a weighted average, where the weights are determined by the prediction score of the individual predictions. We denote the final ensemble model as *Ensemble*.

### Data augmentation

For data augmentation, we crop or pad the x-rays randomly by a maximum of 50 pixels to add robustness for different fields of view. Also, horizontal flipping and random rotation by a maximum of 5 degrees are applied. Furthermore, we blur the x-rays and apply cutout augmentation to improve the generalization of our models. Additionally, Mosaic augmentation and test time augmentation are used exclusively for YoloV5, as only for the YoloV5 models performance improvements are seen.

In test time augmentation, each x-ray is evaluated multiple times for flipped and scaled versions of the x-ray. The predictions are then merged before applying non-maximum-suppression.

**Table 2:** Hyperparameters for training, determined by cross-validation.

Parameter	YoloV5-large	YoloV5-small	Faster-R-CNN	RetinaNet	EfficientDet-D2
Learning rate	8.94e$-$3	1.15e$-$2	1.0e$-$4	1.0e$-$4	1.0e$-$4
Optimizer	SGD	SGD	Adam	Adam	Adam
Batch size	8	16	16	16	16
Epochs	20	20	25	60	20
SWA start	N/A	N/A	20	45	15
SWA epochs	N/A	N/A	5	15	5
Warmup epochs	2.5	2.8	5.0	5.0	5.0
Gradient clipping value	N/A	N/A	3.0	3.0	3.0

For gradient clipping, the gradients’ global norm is clipped to the reported values.

### Imbalanced sampling and nodule generation

To address the challenge of class imbalance, we experiment with oversampling the minority class of our training set to re-balance the mini-batches. During validation, we undersample the majority class. Thereby, we achieve a balanced data set for both training and validation.

As an alternative strategy, we consider generating artificial nodules. To this end, we make use of the nodule generation algorithm^70^ that is also used as a baseline algorithm for the generation track of the Node21 challenge. First, nodules are generated by projecting template nodules from 3D CT scans to the 2D x-ray space by raycasting. Next, the contrast is adjusted to match the intensity range of x-rays. Finally, the nodule is embedded in the chest x-ray at a given position, rotation and scale and the inserted nodule is smoothed by mean filtering. Note that the template nodules, as well as position, rotation and scale of the nodules are taken from the generation track of the Node21 challenge. The generation process is shown in Fig. 1. We randomly sample 1000 x-rays without nodules from the training data and use the generation algorithm to place one or more nodules in these x-rays. Note that the nodule generation is done offline beforehand. As a result, we achieve a balanced data set for training and evaluation without the need for oversampling and thus with a reduced risk for overfitting.

### Transfer learning

We make use of pre-trained model weights to account for the limited training data. We identified the source domain of the pre-trained weights as an important factor for transfer learning in the context of nodule detection. Therefore, we use models that are pre-trained on the VinDR data set^58^ where the model checkpoints originate from the VinBigData Chest X-ray Abnormalities Detection Challenge^71^. This allows us to use pre-trained weights from the same domain as the target domain, i.e. chest x-rays. As alternative, we train the models from scratch without any pre-trained weights and we utilize pretrained models from COCO. Across all models, we keep all layers trainable.

### Training parameters

In general, for fine-tuning hyperparameters, we utilize our held-out validation and test set ($\mathscr {D}_{val}$ and $\mathscr {D}_{add}$) and for broader model decisions we evaluate the algorithms on the experimental test set ($\mathscr {D}_{exp}$). The hyper-parameters differ slightly depending on the model choice. We observe that the metrics on the different data sets ($\mathscr {D}_{add}$ and $\mathscr {D}_{exp}$) are not always congruent which might be caused by the different degrees of imbalances. In cases where no congruent result is found, we choose the solution that works best on the experimental test set, as a smaller domain shift to the final test set is assumed.

For Faster-R-CNN, RetinaNet and EfficientDet-D2, we train a fixed number of epochs, apply stochastic weight averaging (SWA)^61^ and use the last checkpoint for the final prediction. For YoloV5, we validate our models every epoch and early stopping is applied based on the validation set. For all models, cosine annealing^72^ is used as learning rate schedule. A summary of all training parameters is provided in Table 2. Training of our models is performed on NVIDIA RTX 3090 (24GB) and NVIDIA V100 (32GB) graphics cards depending on the model size.

### Runner up solutions

In this section, we briefly describe the algorithms that have been developed by other competitors in their core concepts.

The team that achieved the second place (runner up) chose a similar approach to our solution by ensembling state-of-the-art object detectors, including Faster-R-CNN, YoloV5 and RetinaNet. Furthermore, different methods for sampling data sets to attenuate the class imbalances have been evaluated. Finally, they used a three-fold cross-validation approach where oversampling was used to balance the nodule and background classes.

The team that achieved the third place (second runner up) approached the competition by first pre-training an ensemble of object detectors on slices of CT volumes. After fine-tuning on the node21 data set with oversampling, they used a bagging strategy to aggregate the predictions of their ensemble consisting of Faster-R-CNN and RetinaNet.

## Results

### Evaluation metrics

We use two main performance metrics which are commonly used in the field of nodule detection. First, we evaluate the sample-level performance, i.e., if an x-ray contains a nodule or not by reporting the area under receiver operation characteristic curve (AUROC). The AUROC is calculated by examining the predicted likelihoods of all nodules from each x-ray and filtering for the maximum prediction to derive a sample-level score. If there is no predicted nodule, the sample-level score is set to 0. Second, to evaluate the balance between the sensitivity and false positive predictions on a bounding-box level we report the sensitivity at an average false-positive rate of $25\%$ (FROC$_{25\%}$). To calculate the sensitivity, we count a predicted bounding box as positive if it shares an intersection over union greater than 0.2 with the ground truth bounding box. Otherwise, the prediction is counted as false-positive. Similar to the Node21 competition metric, we also consider a linear combination of the AUROC and FROC$_{25\%}$:

1 $$\begin{aligned} \text {Competition Metric (CM)}=0.75 \cdot \textrm{AUROC} + 0.25 \cdot \textrm{FROC}_{25\%}. \end{aligned}$$

In addition, we provide AUROC and FROC plots and a run-time analysis of the predictions from the evaluated models.

**Table 3:** Performance metrics in percent of the different models and ensembles for different test sets.

Test set	Model	CM	AUROC	FROC$_{25\%}$	FROC$_{50\%}$	Runtime (s/patient)
$\mathscr {D}_{add}$	YoloV5-small	**87.94**	**94.59**	68.00	76.57	0.33
	YoloV5-large	87.12	93.68	67.43	80.00	0.54
	RetinaNet	82.84	90.46	60.00	70.29	0.17
	Faster-R-CNN	84.17	91.28	62.86	79.43	0.17
	EfficientDet-D2	84.36	92.10	61.14	74.86	0.24
	Ensemble	87.77	93.98	**69.14**	**81.14**	1.26
$\mathscr {D}_{exp}$	YoloV5-small	80.99	88.48	**58.51**	**65.03**	N/A
	YoloV5-large	80.00	88.22	55.33	64.11	N/A
	RetinaNet	75.88	85.58	46.77	54.84	N/A
	Faster-R-CNN	76.96	86.38	48.69	58.87	N/A
	EfficientDet-D2	74.53	84.19	45.56	54.03	N/A
	Ensemble	**82.08**	**90.73**	56.12	64.62	N/A
$\mathscr {D}_{final}$	First Place (Ensemble)	**83.90**	**86.79**	**75.24**	**80.00**	N/A
	Runner up	82.75	86.21	72.38	77.14	N/A
	Second runner up	80.11	83.32	70.48	76.19	N/A

$\mathscr {D}_{add}$ denotes our additional validation set for the evaluation of intermediate changes of our approaches and models. $\mathscr {D}_{exp}$ denotes the experimental test set, provided by the challenge hosts for validation on unseen data. $\mathscr {D}_{final}$ denotes the final test set of unseen data to rank the final submissions of all participants. Among with our solution (Ensemble), the top three challenge solutions are reported.

Highest values are in bold.

**Table 4:** Detection performance of the ensemble of all models (**Ensemble**) with and without pre-trained weights and different re-balancing strategies.

Test set	Pretrain	OS	GN	CM	AUC	FROC$_{25\%}$
$\mathscr {D}_{add}$	None			82.64	89.52	61.71
	COCO			85.52 ($\uparrow$3.48 % )	91.93	66.29
	VinDR			86.76 ($\uparrow$4.98 % )	92.83	68.57
	VinDR	$\checkmark$		86.87 ($\uparrow$5.11 % )	92.40	**70.29**
	VinDR		$\checkmark$	**87.36** ($\uparrow$**5.71** % )	**94.19**	66.86
$\mathscr {D}_{exp}$	None			75.43	84.99	46.77
	COCO			79.78 ($\uparrow$5.77 % )	88.50	53.63
	VinDR			80.47 ($\uparrow$6.68 % )	87.94	**58.06**
	VinDR	$\checkmark$		81.45 ($\uparrow$7.98 % )	89.58	57.10
	VinDR		$\checkmark$	**81.87 **($\uparrow$**8.54** % )	**89.86**	57.91

The check marks indicate the use of the respective pre-training or re-balancing strategy. **Pretrain** refers to the dataset used for pre-training of the models, **OS** to oversampling and **GN** to generated nodules. **CM** denotes the competition metric.

Highest values are in bold.

### Comparison of individual models and ensembles

Table 3 shows the results for individual models as well the ensemble for different test sets. The models are trained with pre-trained weights and generated nodules are used to re-balance the training set. For all models, we aggregate the predictions of the individual folds by weighted box fusion^62^. We observe a performance gap across the different test sets. For the held-out test set $\mathscr {D}_{add}$, overall, higher metrics are reported compared to the experimental test set $\mathscr {D}_{exp}$. Regarding $\mathscr {D}_{add}$, YoloV5-small shows high performance, superior to both other individual models and the ensemble of all models. Considering the experimental test set $\mathscr {D}_{exp}$, the ensemble model outperforms all other individual models, including YoloV5-small. By setting a fixed threshold (the median value of all thresholds of the AUROC), we report a true positive rate and true negative rate of 88.57% and 84.00%, at a false positive rate and false negative rate of 13.14% and 13.71% respectively for the ensemble. Notably, the inference run-time of the evaluated models varies strongly. Comparing YoloV5-small and the ensemble, improving the competition metric by 1.3% results in a run-time increase of 280%.

**Figure 4:**
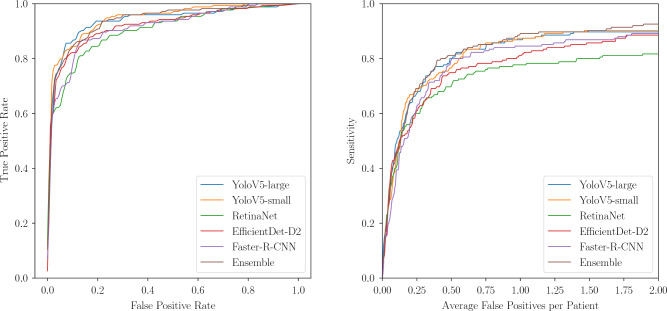
Left: AUROC of all individual models and their ensemble, evaluated on $\mathscr {D}_{add}$. Right: FROC of all individual models and their ensemble, capped at an average of two false positives per patient.

For a fine-grained comparison of detection performances regarding $\mathscr {D}_{add}$, the AUROC and FROC are plotted in Fig. 4. Furthermore, in Fig. 3, exemplary predictions of the Ensemble are shown. It can be observed that false positive predictions often occur at locations of overlapping structures.

In Table 3, final challenge results are shown for the top three approaches. While the runner up solution shows competitive AUROC scores, it is clearly outperformed by our Ensemble solution considering FROC-based metrics, indicating a better detection performance.

### Ablation study: class imbalance and pretrained weights

Table 4 shows ablations for the ensemble of all models. We compare training from scratch and the use of transfer learning with pre-trained weights from different domains. We tackle the class imbalance with oversampling (OS) or generated nodules (GN). Training the network from scratch results in poor performance compared to models that use pre-trained weights. Comparing weigths from COCO and VinDR, models that are trained with VinDR show superior detection performance.

Considering the different strategies for addressing the class imbalance, nodule generation shows improved detection performance compared to simple oversampling for both data sets, regarding the AUROC and Competition Metric.

## Discussion

Automated nodule detection in chest x-rays is of high clinical importance. Although deep learning algorithms show high performance for general purpose object detection tasks on benchmark data sets, their application to real-world, clinical problems such as nodule detection is challenging. In this work, we systematically identify these challenges and study approaches to tackle them. Thereby, we investigate which source data set is best suited for transfer learning and study different strategies to re-balance the imbalanced training data. Furthermore, we systematically compare four state-of-the-art deep learning models for object detection. Finally, we present a highly effective deep learning approach by aggregating complementary model predictions from different object detectors.

Overall, we observe superior performance when evaluating on the held-out test set compared to the experimental- and final test set. This indicates that the models tend to overfit to the domain of the training data set. Another reason for this is seen in the annotation process of the test sets provided by the Node21 challenge. The annotations are derived with the help of CT scans in which nodules can be detected much easier. Since for the annotation of the training data, no CT scans are used, detecting all nodules from the test set is challenging as very subtle nodules may exist that only can be seen in CT scans. This highlights the importance of data annotation and represents a drawback of the training data.

Our results show that the individual models Faster-R-CNN, RetinaNet and EfficientDet-D2 show similar performance. In contrast, both Yolo versions show superior performance to other individual models. We assume that the pipeline of Yolo which includes pre-processing, data augmentation and anchor generation techniques that are highly optimized for the Yolo network architecture leads to a strong detection performance. In contrast, for the other models, although data augmentation is applied, the augmentation policies are not directly optimized to a certain model. We note that in comparison to all other models RetinaNet is initialized from COCO weights instead of the VinDR weights data set as no checkpoints are available for RetinaNet which hinders a direct and fair comparison.

By ensembling the individual models, we can further improve the performance compared to the best individual model YoloV5-small regarding the competition metric on the unknown experimental test set. For a fixed threshold, the ensemble shows a high true positive rate while preserving a small number of overseen nodules which is important, e.g. for pre-screening purposes. While the performance boost of the ensemble on the experimental test set is moderate compared to the rise in computational costs, we argue that since even small improvements are of high relevance, the additional costs in run-time are acceptable.

Using an ensemble of different models is often considered in practical applications where high-performance on unseen data is important as ensembling has shown to be a good strategy to combine strong features from individual algorithms^50,51^. Even though a single model might outperform an ensemble on a certain data set, it is unrealistic to train a single model that generalizes to all possible variations in data. Therefore, on average, ensembles are assumed to be a more general solution^52^. This can be observed considering Table 3. While for our held-out set $\mathscr {D}_{add}$, the individual YoloV5 model shows a higher CM in comparison to the ensemble model, for the unknown set $\mathscr {D}_{exp}$ the ensemble is superior. Furthermore, although all individual models appear to perform poorly compared to YoloV5-small regarding the FROC analysis (Fig. 4), the ensemble benefits from their contributions as the individual models show different characteristics at different observation points.

We believe that combining different algorithms with different detection characteristics and input sizes generalizes well to different nodule sizes and appearances, to different x-ray examination protocols and to varying resolutions.

Hence, we choose the ensemble of all models for our final solution to become agnostic to certain characteristics in the training and test data. This is identified as an important property of the final solution for the Node21 challenge, as it is evaluated on an unseen data set $\mathscr {D}_{final}$.

Pre-training shows notable performance improvements across all metrics. Initializing the models with weights that are trained on generic images from COCO already improves the models final predictions. We assume that the CNNs benefit from the weights of the early layers in the feature extraction backbones that are trained to detect edges and different shapes in the images. Thereby, the risk for overfitting is reduced and faster convergence can be achieved^54^. Using data from the target domain, i.e., chest x-rays, for transfer learning further improves the performance. This is not surprising as now the pre-trained filters of the CNNs are already specialized to the task of object detection in chest x-rays and we assume that during fine-tuning, later layers of the models are also reused and only require subtle adaptions. Overall, we identify pre-training as an important strategy to improve the detection performance and highlight the positive effect of using image data from a similar domain as the target data set for pre-training.

We evaluate different strategies to encounter the class imbalance in the data. We find that it is important to re-balance the training data. However, there is no significant result on whether nodule generation or simple oversampling is superior in general. Comparing both strategies for the task at hand, nodule generation is seen beneficial as the risk of overfitting is reduced^73^. Furthermore, as the generated nodules are barely visible by design, we believe that this helps to make the models more sensitive to subtle nodules that might appear more clear in the CT scans that are used as additional modality only for test set annotations.

Comparing our approach to the runner up and second runner up solutions in the node21 competition, our solution consistently outperforms the other methods, particularly when considering the FROC-based localization metrics. Both competing solutions chose a similar approach for the competition, namely an ensemble of recent object detection models. However, we believe that our systematic evaluation of strategies to address challenges for the task at hand have considerably improved the performance of our solution. First, as shown in this study, clear benefits can be observed for pre-training across all models and test sets which is not part of the runner up solution. While the second runner up achieved performance improvements when pre-training on CT data, their model ensemble did not include Yolo-based models that have shown to perform best within our study regarding the node21 competition. Second, even though only moderate performance improvements can be achieved by nodule generation, we believe that this strategy has led to increased generalization as oversampling which is done in both competing solutions comes with a risk for overfitting.

In conclusion, by conducting a careful analysis and by addressing identified challenges, we present a high performing deep learning pipeline for the detection of nodules in chest x-rays. We prove the effectiveness and robustness of our strategy by winning the detection track of the Node21 challenge with a relative improvement of 1.4 % to the second-best submission. To build an approach that achieves state-of-the-art performance, the use of pre-trained weights from the same image domain, re-balancing of the training set, and ensembling of various model architectures are seen as key strategies. Furthermore, the careful design of validation and test splits, hyper-parameter tuning and data augmentation are seen as requirements for a robust deep learning-based solution.

While our method outperforms all competing solutions, it is essential to acknowledge that further studies are needed to evaluate the clinical utility of the proposed solution. The performance of the system in a clinical setting will depend on various factors, such as the prevalence of nodules in the patient population, the experience of the radiologist, and the available resources for follow-up procedures.

We also recognize the limitations of the high false positive rate, as false positives can lead to unnecessary follow-up CT scans, resulting in patient anxiety, increased radiation exposure and costs. Therefore, radiologists would need to carefully review the results of CAD systems, as also noted in clinical validation studies of commercial systems^32,34,74^. To reduce the manual filtering of false-positive predictions from CAD systems, further research is needed to increase sensitivity while reducing the false positive rate to an acceptable level. One possible solution is the use of two-planar projections that include the lateral view. We hypothesize that this could prevent the models from interpreting overlapping structures or blood vessels as nodules and thus improve the detection performance of nodules in chest x-rays while reducing false-positive findings. We believe that further exploration of this approach may be a promising direction for future research in the field of chest x-ray nodule detection.

## Data Availability

The data sets that are used in this work are publicly available via the Node21 Competition https://zenodo.org/record/5548363#.Y4TNvX2ZNQ4. The code for our approach, model checkpoints and a Docker Image are available at https://github.com/FinnBehrendt/node21-submit.
